# A head-to-head comparison of humoral and cellular immune responses of five COVID-19 vaccines in adults in China

**DOI:** 10.3389/fimmu.2024.1455730

**Published:** 2024-08-21

**Authors:** Xu Han, Hongxing Pan, Pengfei Jin, Mingwei Wei, Siyue Jia, Wenjuan Wang, Kai Chu, Shuyu Gao, Li Zhou, Jingxin Li, Fengcai Zhu

**Affiliations:** ^1^ National Vaccine Innovation Platform, School of Public Health, Nanjing Medical University, Nanjing, China; ^2^ National Health Commission Key Laboratory of Enteric Pathogenic Microbiology, Jiangsu Provincial Center for Disease Control and Prevention, Nanjing, China

**Keywords:** head-to-head, humoral immune, cellular immune, COVID-19 vaccines, SARS-COV-2 variant

## Abstract

**Introduction:**

Various COVID-19 vaccine trials have shown that vaccines can successfully prevent symptomatic cases of COVID-19 and death. Head-to-head comparisons help to better understand the immune response characteristics of different COVID-19 vaccines in humans.

**Methods:**

We randomly selected 20 participants from each of five ongoing Phase II trials of COVID-19 vaccines. Here, SARS-CoV 2-specific immune responses to DNA vaccine (INO-4800), mRNA vaccine (BNT162b2), Adenovirus-vectored vaccine (CONVIDECIA), Protein subunit vaccine (Recombinant COVID- 19 Vaccine (Sf9 Cells)), Inactivated Vaccine (KCONVAC) were examined longitudinally in healthy adults between Jan 15, 2021 and July 5, 2021 for 6 months. RBD-IgG titres were detected by ELISA, neutralising antibody titer were detected by pseudoviral neutralization and immune cell response were detected by flow cytometry.

**Results:**

At the first visit (V1), 100% of individuals who received the BNT162b2, CONVIDECIA, or KCONVAC vaccines experienced seroconversion of neutralizing and binding antibodies in the serum. Except for the Recombinant COVID-19 Vaccine (Sf9 Cells) vaccine having the highest neutralizing antibody GMT at the second visit (although there was no statistically significant difference in geometric mean titers between V1 and V2), the rest of the vaccines had the highest levels of binding antibodies and neutralizing antibodies at V1. The neutralizing antibodies GMT of all vaccines showed a significant decrease at V3 compared to V1. The neutralizing antibody GMT against the omicron variant of all vaccines at V1 showed a significant decrease compared to the wild strain. We observed statistically significant differences in Tcm cells and RBD-specific memory B cells among various vaccines.

**Discussion:**

BNT162b2 (mRNA vaccine) exhibits the highest antibody levels among the five vaccines evaluated, regardless of whether the target is the wild-type virus or its variants. However, its cellular immune response may be weaker compared to CONVIDECIA (adenovirus type 5 vector vaccine).

## 1 Introduction

The accelerated development of COVID-19 vaccines has created a stage for multiple vaccines to compete simultaneously. Although these vaccines have individually reported better immunogenicity or significant protective efficacy in different clinical trials, the immunogenicity characteristics induced by vaccines may vary depending on the vaccine type. However, obtaining data under different clinical trial conditions or testing in different laboratories makes cross-comparison of data challenging. Furthermore, most of the immunogenicity data reported by these vaccines are for the Wild strain, and there is little data on neutralization efficacy against various emerging variants (1–6).

Our study established 5 different vaccines immunogenicity and immune persistence sub-cohorts based on the clinical research cohorts of the INO-4800 vaccine (NCT04336410), BNT162b2 vaccine (NCT04649021), CONVIDECIA vaccine (NCT04566770), Recombinant COVID-19 Vaccine (Sf9 Cells) (NCT04640402), and KCONVAC vaccine (ChiCTR2000039462). INO-4800 is a DNA vaccine targeting the spike protein of the SARS-CoV-2, BNT162b2 is an mRNA vaccine encoding the full-length spike protein, CONVIDECIA is an Ad5 viral vector vaccine encoding the SARS-CoV-2 spike protein, the Recombinant COVID-19 Vaccine is a recombinant subunit vaccine carrying the receptor-binding domain protein of the SARS-CoV-2 spike protein, and KCONVAC is an inactivated whole virus vaccine for the SARS-CoV-2. The antigens of these five vaccines are all designed against the wild strain of the SARS-CoV-2.

For the first time, a head-to-head comparison of the antibody response levels and decay patterns induced by these vaccines post-immunization was carried out. We also evaluated the cross-reactivity of antibody levels generated by these vaccines against variant strains, as well as changes in post-immunization immune cells.

## 2 Methods

### 2.1 Study design, participants and procedures

The INO-4800 vaccine trial included a total of 160 adult subjects in the 2 mg dose group, with a randomization ratio of 3:1 between the vaccine group and the placebo group. The BNT162b2 vaccine’s Phase II clinical trial ultimately enrolled 720 subjects in the vaccine group and 240 subjects in the placebo group, with a randomization ratio of 3:1. The CONVIDECIA study ultimately included 250 participants, with a randomization ratio of 1:1 between the low-dose group and the placebo group. The Recombinant COVID-19 Vaccine (Sf9 Cells) immunization schedule of 0-14-28 days involved 120 subjects, who were randomly assigned in a 5:1 ratio to receive either the vaccine or placebo. The KCONVAC vaccine Phase II clinical trial for the age group of 18-59 years included high-dose, medium-dose, and placebo groups with a total ratio of 2:2:1. For detailed information, please refer to the registration information for each vaccine on the Clinical Trials or Chinese Clinical Trial Registry Registration websites. We have sequentially selected 20 individuals from each of the five immunization cohorts INO-4800 (delivered intradermally+electroporation), BNT162b2 (intramuscular injection, IM), CONVIDECIA (intramuscular injection, IM), Recombinant COVID- 19 Vaccine (Sf9 Cells) (intramuscular injection, IM), KCONVAC (intramuscular injection, IM). The sampling was conducted in a blinded manner, and the sampling proportion was consistent with the proportion of the experimental group and the control group set in the original sample. However, due to special reasons (blinded state), the final samples taken in the inactivated vaccine (KCONVAC) group comprised two dose groups and the control group. Ultimately, in this paper, we used the 5μg dose group (the dose group used in the phase 3 clinical trial) (7 individuals) and the control group (4 individuals) as the subjects for analysis. Recruitment of healthy male or female participants, aged 18-59 years, who have not received the COVID-19 vaccine. The investigators examined the participants’ vaccination records and medical histories. The major criteria for inclusion were axillary temperature ≤37°C, anti-SARS-CoV-2 IgM and IgG in serum are negative, and previous medical history and physical examination indicating healthy condition. Exclusion criteria include a history of SARS-CoV-2 infection or COVID-19 epidemiological exposure, a previous vaccination against COVID-19, a severe acute allergic reaction to the any ingredient of vaccine, and other serious medical conditions. (Appendix 1 for complete inclusion and exclusion criteria). Blood samples were collected from all participants before vaccination and at 1 month, 3 months, and 6 months after vaccination. Serum and peripheral blood mononuclear cells (PBMCs) samples were collected and stored at -80°C/liquid nitrogen until use. A total of 91 participants completed three follow-up visits. The protocol and informed consent were reviewed and approved by the Ethics Committee of the Jiangsu Center for Disease Control and Prevention (JSJK2021-A010-01) before the launch of the trial, and no changes were made to the protocol after the study began.

### 2.2 Enzyme-linked immunosorbent assay

Serum samples were diluted at 1:200 to measure the RBD-specific ELISA antibody response. Commercial anti-SARS-CoV-2 RBD IgG ELISA kit was used (RM4143; Vazyme Biotech Co.,Ltd). The OD value=OD450-OD630. Cut-off value = 0.17+ mean value of negative control well, the cut-off value was finally determined as 0.22. For the purpose of calculation, antibody concentration values below the lower limit of quantification (LLOQ=624) were replaced with 0.5 x LLOQ and ultimately set at 312.

### 2.3 SARS-CoV-2 pseudoviral neutralization assay

Sera from vaccinated participants were heat-inactivated and serially diluted 3-fold starting at 1:30 dilution for the SARS-CoV-2 pseudoviral neutralization assay. The diluted sera were mixed with different SARS-CoV-2 pseudovirus using HIV-1 pseudovirus system expressing the spike glycoprotein of SARS-CoV-2 strains, including Ancestral; Beta B.1.351; Gamma P.1; Delta B.1.617.2; Omicron B.1.1.529 at 2*10^4 TCID50/ml; 50ul/well; in 96-well plates for 60 min at room temperature, respectively. After incubation, sera-pseudovirus mixture was added to HEK293-ACE2 (50ul/well, 0.4*10^6 cells/mL) and allowed to incubate in a standard incubator (37% humidity, 5% CO2) for 48 h. After 48h incubation in a 5% CO2 environment at 37°C, the culture supernatant was aspirated gently to leave 100 μl in each well; then, 100 μl of luciferase substrate (Vazyme, DD1201-01) was added to each well. Three min after incubation at room temperature, 100 μl of lysate was transferred to white solid 96-well plates for the detection of relative luminescence unit (RLU) values. Neutralization titers (ID50) were calculated using GraphPad Prism 8 and defined as the reciprocal serum dilution at which RLU were reduced by 50% compared to RLU in virus control wells after subtraction of background RLU in cell control wells.

### 2.4 Flow cytometry analysis experiments of T cells and B cells

Analysis of the expression levels of naive T cell (Tn), effector T cell (Te), effector memory T cell (TEM), memory stem T cell (Tscm), central memory T cells (Tcm), memory B, plasmacyte, and RBD-specific B cell in PBMC samples collected at 1-month post-vaccination. Flow cytometry gating strategy for identification of T and B cells are shown in **Supplementary Figure S4**. Resuscitate PBMC cells from different vaccine recipients, perform viability staining using Fixable Viability Stain, block Fc receptors with Human BD Fc Block, and conduct surface flow antibody staining using the following panel of antibodies: PE-CF594 Mouse Anti-Human CD20(2H7), PE Mouse Anti-Human IgG(G18-145), PerCP-Cy5.5 Mouse Anti-Human IgD(IA6-2), BV605 Mouse Anti-Human CD38(HB7), Alexa Fluor 700 Mouse Anti-Human CD4(RPA-T4), BV510 Mouse Anti-Human CD8(RPA-T8), BD Horizon™ BV786 Rat Anti-Human CCR7 (CD197), PE-Cy5 Mouse Anti-Human CD45RO, BUV395 Mouse Anti-Human CD45RA(HI100), PE-Cy7 Mouse Anti-Human CD95(DX2), BD OptiBuild™ BV711 Mouse Anti-Human CD62L, BV421 Mouse Anti-Human CD27(M-T271). All antibodies were purchased from BD. Staining was also carried out using a self-labeled RBD antibody (WT-RBD-488) specific to the wild-type strain of the SARS-COV-2 (utilizing YF^®^488 (5) succinimidyl ester for RBD antigen labeling). Analysis was performed using a BD FACSAriaTM Fusion flow cytometer.

### 2.5 Statistical analysis

The neutralizing antibody titers or concentrations were calculated with the two-sided 95% CIs, based on the t-distribution of the log-transformed titers or concentrations, and were then back-transformed to the original scale. When calculating GMT/GMC, SARS-CoV-2 antibodies below the limit of detection were assigned half of the detection threshold. Participants were considered seroconversion if the baseline titers or concentrations were undetectable but became detectable after vaccination or increased at least four-fold compared to detectable baseline levels. Normality of the data was assessed using the Shapiro-Wilk test. Analysis of normally distributed data among groups was conducted using analysis of variance, while non-normally distributed data were analyzed using the Wilcoxon rank-sum test. Categorical data, such as seroconversion, were analyzed with the χ2 test or Fisher’s exact test. The non-parametric Spearman correlation analysis was conducted between the specific RBD-binding antibody concentration detected by ELISA and the neutralizing antibody titers against the SARS-CoV-2 wild-type pseudovirus. Statistical analysis was performed using SAS (version 9.4) or GraphPad Prism (version 8.0.1).

## 3 Results

In order to compare the development of immunogenicity, we recruited subjects who have been vaccinated with INO-4800, BNT162b2, BNT162b2, Recombinant COVID-19 Vaccine (Sf9 Cells), and KCONVAC vaccines. The characteristics of the donor pool are shown in **Table 1**. All five vaccine groups were similar in their distribution of gender, age, and baseline immunogenicity (**Table 1**). Blood samples were collected at multiple time points, and plasma and peripheral blood mononuclear cells (PBMC) were separated and preserved. For example, sampling time points for INO-4800 were pre-vaccination (V0), and then three sampling time points after immunization (V1 to V3), counting days after the last immunization: V1 at 36 ± 2 days, V2 at 95 ± 2 days and V3 at 184 ± 2 days. Both cohorts of vaccinees (Recombinant COVID-19 Vaccine (Sf9 Cells), KCONVAC) received three doses of the vaccine, approximately 14 and 28 days apart, respectively. Among the cohorts of vaccinees (INO-4800, BNT162b2, CONVIDECIA), two doses of the vaccine were administered, with intervals of approximately 28, 21, and 56 days, respectively. The median age of the participants across the groups was 43.5 to 50 years old. Prior to vaccination, the titers of neutralizing antibodies against the pseudovirus and anti-RBD IgG were all negative.

**Table 1:** Demographic characteristics, baseline immunization characteristics .

Characteristic	INO-4800	BNT162b2	CONVIDECIA	Recombinant COVID- 19 Vaccine (Sf9 Cells)	KCONVAC	Placebo
**Donors, n**	12	17	13	18	7	24
Gender,n(%)						
Male	8(66.7%)	8(42.1%)	5(38.5%)	10(55.6%)	5(71.4%)	10(41.7%)
Female	4(33.3%)	9(52.9%)	8(61.5%)	8(44.4%)	2(28.6%)	14(58.3%)
Age						
Median (IQR)	48.5(34-49)	50(45-52)	48(42.5-53.5)	44.5(40.5-48.5)	48(38-52)	43.5(37-50)
Time from the last dose (Days)						
V1	36 ± 2	32 ± 2	55	39 ± 3	57	43.5 ± 13.5
V2	95 ± 2	91 ± 2	124	97 ± 3	112	106.5 ± 17.5
V3	184 ± 2	174 ± 2	212	197 ± 3	188	192 ± 20
V0 RBD-IgG						
GMT	5(5.0-5.0)					
V0 Neutralising antibody to pseudovirus(WT)						
GMT	15.0(15.0-15.0)					
Immunization regimens						
	0, 28	0, 21	0, 56	0, 14, 28	0, 28, 56	
Dosage						
	2.0 mg	30ug	5×10^10^vp	40μg	5ug	–
Type of vaccine						
	DNA	mRNA	Viral vector	Protein subunit	inactivated virus	
Clinical Trials.gov Identifier:/Chinese Clinical Trial Registry Registration number:						
	NCT04336410	NCT04649021	NCT04566770	NCT04640402	ChiCTR2000039462	

### 3.1 Antibody magnitude and durability against wild-type strain elicited by different vaccine

**Figure 1:**
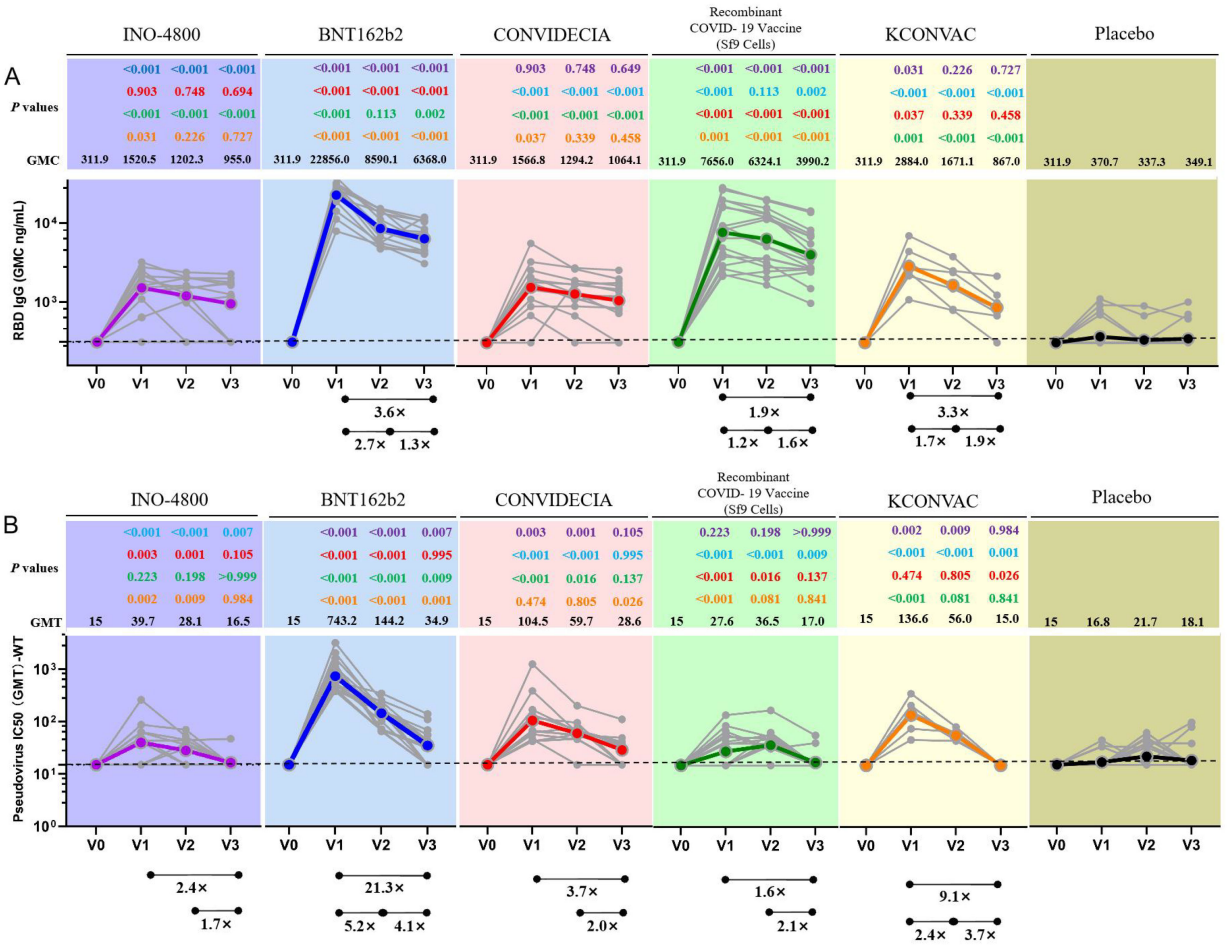
Antibodies elicited by INO-4800, BNT162b2, CONVIDECIA, Recombinant COVID-19 Vaccine (Sf9 Cells) and KCONVAC COVID-19 vaccine. **(A)** Comparison of longitudinal SARS-CoV-2 RBD IgG concentration **(B)** SARS-CoV-2 pseudovirus neutralizing titers (WT) from all donors to the INO-4800 (purple), BNT162b2 (blue), CONVIDECIA (red), Recombinant COVID-19 Vaccine (Sf9 Cells) (green), KCONVAC (yellow) and Placebo (black) in full immunization four sampling time points. Individual subjects are show as gray symbols with connecting lines for longitudinal samples. Geometric means are shown in thick colored lines. Dotted lines indicate the limit of quantification (LOQ). *p* values show differences between each time point between the different vaccines, color-coded per comparison based on the vaccine compared. GMT: geometric mean titers. The bottom bar represents a fold change indicating a statistically significant difference in GMT between two time points.

For INO-4800, at the first visit (V1) after full immunization, 92.2% of vaccinees had detectable anti-RBD-IgG concentration and 66.7% of vaccinees had detectable neutralizing antibody titers (**Table 2**). The anti-RBD-IgG GMC and neutralizing antibody GMT peaked at V1, reaching 1520.5 (95% CI: 1035.1-2233.6) and 39.7 (95% CI: 22.8-69.1), respectively. 67% and 6.2% of INO-4800 recipients remained seroconversion for anti-RBD-IgG and neutralizing antibodies at V3. There was a notable 2.4-fold decrease from peak(V1) to trough(V3) for neutralizing antibodies (**Figures 1A, B**).

**Table 2:** Antibody responses at V1, V2 and V3 post-administration of the last vaccine dose.

Items	INO-4800	BNT162b2	CONVIDECIA	Recombinant COVID- 19 Vaccine (Sf9 Cells)	KCONVAC	Placebo
**N**	12	17	13	18	7	24
V1(30-57 days after full immunization)						
Anti-RBD-IgG						
Seroconversion	11(92.2,86.6-97.4)	17(100,96.0-100)	12(92.2,86.6-97.4)	18(100,96.0-100)	7(100,96.0-100)	4(17.0,9.5-24.5)
GMC	1520.5(1035.1-2233.6)	22856.0(18793.2-27733.2)	1566.8(1047.1-2344.2)	7656.0(5116.8-11455.1)	2884.0(1832.3-4539.4)	370.7(315.5-435.5)
Pseudovirus neutralising (WT)						
Seroconversion	8(66.7,34.9-90.1)	17(100,80.5-100)	13(100,75.3-100)	8(44.4,21.5-69.2)	7(100,59.0-100)	3(12.5,2.7-32.4)
GMT	39.7(22.8-69.1)	743.2(531.7-1038.7)	104.5(57.8-189.1)	27.6(18.9-40.1)	136.6(73.9-252.6)	16.8(14.7-19.1)
V2(89-124 days after full immunization)						
Anti-RBD-IgG						
Seroconversion	10(83.0,75.7-90.5)	17(100,96.0-100)	12(92.2,86.6-97.4)	18(100,96.0-100)	7(100,96.0-100)	2(8.0,2.6-13.4)
GMC	1202.3(809.1-1790.6)	8590.1 (7046.9-10495.4)	1294.2(935.4-1790.6)	6324.1(4295.4-9289.7)	1671.1 (1059.3-2636.3)	337.3(302.7-375.8)
Pseudovirus neutralising (WT)						
Seroconversion	7(58.3,27.7-84.8)	17(100,80.5-100)	12(92.3,64.0-99.8)	14(77.8,52.4-93.6)	7(100,59.0-100)	9(37.5,18.8-59.4)
GMT	28.1(19.3-40.7)	144.2(111.7-186.3)	59.7(42.4-83.9)	36.5(26.9-49.5)	56(45.4-69.1)	21.7(17.5-26.9)
V3(172-212 days after full immunization)						
Anti-RBD-IgG						
Seroconversion	8(67.0,57.6-76.4)	17(100,96.0-100)	11(85.0,77.9-92.1)	18(100,96.0-100)	6(100,96.0-100)	3(12.9,6.2-19.5)
GMC	955.0(586.1-1559.6)	6368.0(5236.0-7762.5)	1064.1(739.6-1527.6)	3990.2(2844.5-5597.6)	867.0(547.0-1374.0)	349.1(307.6-396.3)
Pseudovirus neutralising (WT)						
Seroconversion	1(6.2,0.2-30.2)	12(70.6,44.0-89.7)	8(61.5,31.6-86.1)	2(11.1,1.4-34.7)	0	3(12.5,2.7-32.4)
GMT	16.5(13.4-20.3)	34.9(24.5-49.7)	28.6(19.7-41.5)	17.0(14.1-20.5)	15.0(15.0-15.0)	18.1(14.5-22.5)

GMC, Geometric mean concentration; GMT, Geometric mean titer; CI, Confidence interval; N, Number of participants included in each group for the immunogenicity analysis; RBD-IgG, Antibody directed against the receptor-binding domain. Data consist of the GMC with a 95% CI for GMC, the number of participants who tested seropositive expressed as a % with a 95% CI for seroconversion, and the GMT with a 95% CI for GMT.

For BNT162b2, at the first visit (V1), 100% of vaccinees had detectable RBD-IgG and neutralizing antibodies (**Table 2**). At the second visit (V2), the neutralizing antibody titer decreased by 5.2 times compared to V1 (743.2 (531.7-1038.7) vs 144.2 (111.7-186.3)), and the binding antibody decreased by 2.7 times (22856.0 (18793.2-27733.2) vs 8590.1 (7046.9-10495.4)) (**Table 2**). 100% of BNT162b2 recipients remained positive for RBD IgG, and 70.6% for neutralizing antibodies at V3 (**Table 2**). From V1 to V3, GMC of RBD-IgG and neutralizing antibody GMT decreased by 3.6-fold, and 21.3-fold, respectively. The neutralizing antibody GMT in BNT162b2 recipients was higher than that in INO-4800 recipients by 18.7-fold (*P* < 0.001), 5.1-fold (*P* < 0.001), and 2.1-fold (*P* = 0.007) at the V1, V2, and V3 time points, respectively (**Figure 1**). The gap was gradually narrowing. What was clear is that, except for V3 compared to the CONVIDECIA vaccine, at all other time points, the GMT of neutralizing antibodies for BNT162b2 is significantly higher than the other groups (**Figure 1**).

For CONVIDECIA, similar to other vaccines, peak levels were observed at post-vaccination V1, with 92.2% and 100% of subjects showing seroconversion for anti-RBD-IgG antibodies and neutralizing antibodies, respectively (**Table 2**). The decline in binding antibodies from V1 to V3 showed no statistically significant difference, while the 3.7-fold decrease in neutralizing antibodies from V1 to V3 was statistically significant (104.5(57.8-189.1) VS 28.6(19.7-41.5), *P*<0.05) (**Table 2**). At V1, the GMT of neutralizing antibodies in the CONVIDECIA vaccine was significantly higher than in the INO-4800(*P* < 0.001) and Recombinant COVID-19 Vaccine (Sf9 Cells) vaccines (*P* < 0.001), but significantly lower than in the BNT162b2 vaccine (*P* < 0.001) (**Figure 1**).

For the Recombinant COVID-19 Vaccine (Sf9 Cells), the concentration of anti-RBD-IgG after vaccination at V1 was quite high, second only to the levels at V1 for the BNT162b2 vaccine. The GMC of anti-RBD-IgG significantly decreased at V2 and V3 (7656.0 (5116.8-11455.1) vs. 6324.1 (4295.4-9289.7) vs. 3990.2 (2844.5-5597.6), *P*<0.05) (**Figure 1**). It was quite peculiar that, although the anti-RBD-IgG GMC levels were relatively high at all time points, the GMT of neutralizing antibodies was very low, almost identical to the neutralizing antibody GMT of the INO-4800 vaccine at each time point, with no statistically significant differences observed (*P* = 0.223, *P* = 0.198, *P* > 0.999) (**Figure 1**).

For KCONVAC immunization, 100% of vaccinees had detectable RBD IgG at V1, V2, V3 and neutralizing antibodies at V1 and V2 (**Table 2**). KCONVAC neutralizing antibody GMT peaked at V1 (GMT 136.6), but that peak was still 5.4-fold lower than the BNT162b2 peak (GMT 743.2). From V1 to V3, neutralizing antibody GMT decreased by 9.1-fold (**Table 2**). Overall, all five vaccines induced significant antibody responses, with serum conversion rates of over 92.2% at V1, compared to a serum conversion rate of 17% in the placebo group (**Table 2**). The serum conversion rates of anti-RBD-IgG for the BNT162b2, CONVIDECIA, and KCONVAC COVID-19 vaccines at the three visit points (V1, V2, V3) were all 100% (**Table 2**). Except for the Recombinant COVID-19 Vaccine (Sf9 Cells), the levels of anti-RBD-IgG and neutralizing antibodies for the other four SARS-CoV-2 vaccines consistently showed good consistency in terms of increase and decrease (**Figure 1**). Among the 5 COVID-19 vaccines, the mRNA vaccine (BNT162b2) exhibited the best performance, with the highest anti-RBD-IgG GMC and pseudovirus neutralizing titer among the 5 vaccines at V1, at 22856.0 (18793.2-27733.2) and 743.2 (531.7-1038.7), respectively (**Figure 1**). Antibody titers to INO-4800, BNT162b2, CONVIDECIA, Recombinant COVID-19 Vaccine (Sf9 Cells) and KCONVAC changed substantially over the 6 months of observation, with different patterns seen for the different platforms.

### 3.2 Correlation analysis of SARS-CoV-2 (WT) pseudovirus neutralization test (GMT) and ELISA (RBD IgG GMC)

When comparing the correlation between neutralizing antibody titers of each vaccine and RBD IgG concentration at each time, only INO-4800 vaccine showed a good correlation at v2, while other vaccines and time points showed no correlation. r and P values were shown in (**Supplementary Figures S1A-C**).

When correlations were analyzed between neutralizing antibody titers and anti-RBD-IgG GMC without distinguishing vaccine species, there was a correlation between detecting RBD IgG titres by ELISA and neutralizing antibody titers by pseudovirus neutralizing method. r and *P* values at the time points V1,V2 and V3 are(r=0.6843, p<0.0001), (r=0.6082, p<0.0001) and (r=0.2474, p=0.018). Overall, we found that neutralizing antibody titers and RBD IgG titres of different vaccine platforms were not necessarily correlated, but the variation was generally consistent with the same trend.

### 3.3 Comparison of neutralization antibody levels of different vaccines against different variant (WT, Beta, Gamma and Epsilon)

Obviously, the neutralization ability of any vaccine against Beta variant (B.1.351) was weak at any visit site, and the neutralization ability of BNT162b2 against Beta variant (B.1.351) decreased by 19.03-fold compared with WT strain at V1 time point (P<0.001). The neutralization ability of KCONVAC against Beta variant (B.1.351) at V1 was 8.18-fold lower than that of WT strain (P<0.001) (**Supplementary Figures S2**, **S3**). The Recombinant COVID-19 Vaccine (Sf9 Cells) had weak neutralizing ability against WT, Beta, Gamma, and Epsilon strain (**Supplementary Figure S2**). Neutralizing antibody GMT of INO-4800 Vaccine and Recombinant COVID-19 Vaccine (Sf9 Cells) vaccine against all four strains was near the detection line at V1 and V2. BNT162b2 had the highest pseudovirus neutralization GMT at all time points and variants (**Supplementary Figure S3**). The GMT decline factor of each vaccine variant relative to the WT strain was shown in **Supplementary Figures S2**, **S3**.

### 3.4 Comparison of neutralization antibody levels of different vaccines against Delta and Omicron variants

**Figure 2:**
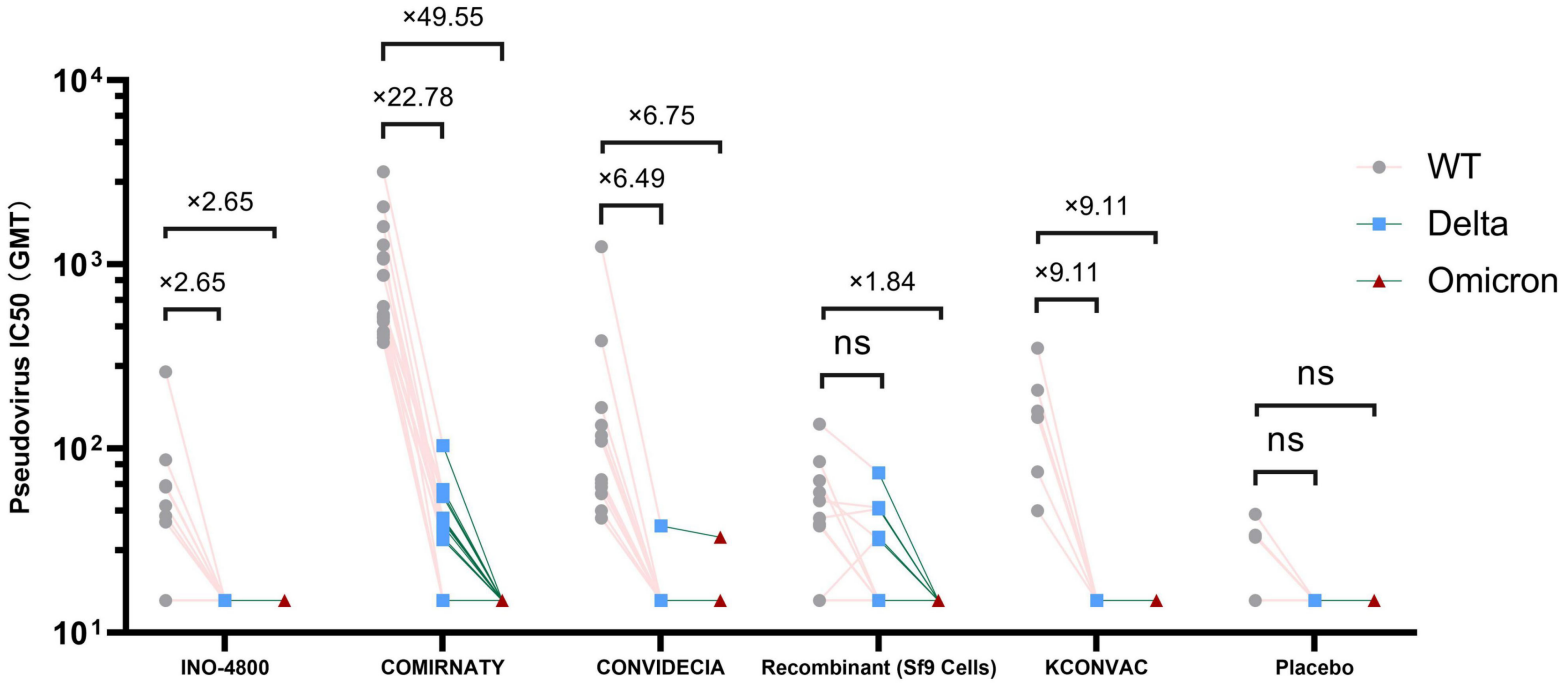
Antibody titres of SARS-CoV-2 pseudovirus (Delta and Omicron) induced by vaccine or placebo. NS stands for P-value ≥0.5.

Pseudovirus neutralization data showed that at V1, the neutralization activity of the vaccine against delta and omicron variants was significantly reduced compared to the wild strains, and even seroconversion changed from 100% to 0%.(**Table 3**, **Figure 2**). Seroconversion of BNT162b2, CONVIDECIA and recombinant COVID-19 vaccine (Sf9 cells) to delta variant strains was 12 (70.6,46.6-94.7), 1(8.3, -10-26.7) and 5(27.8,4.9-50.7), respectively. The GMT of neutralizing antibodies against delta variants of the three vaccines were 32.63, 16.11 and 20.3, respectively (**Table 3**, **Figure 2**). Only CONVIDECIA had 7.7% seroconversion against omicron, and the pseudovirus neutralization titers of the other vaccines against omicron were all below the detection limit.

**Table 3:** Neutralizing antibody levels of pseudovirus (Delta and Omicron) at V1.

Items	INO-4800	BNT162b2	CONVIDECIA	Recombinant COVID- 19 Vaccine (Sf9 Cells)	KCONVAC	Placebo
N	12	17	13	18	7	24
WT						
Seroconversion	8(66.7,34.9-90.1)	17(100,80.5-100)	13(100,75.3-100)	8(44.4,21.5-69.2)	7(100,59.0-100)	3(12.5,2.7-32.4)
GMT	39.7(22.8-69.1)	743.2(531.7-1038.7)	104.5(57.8-189.1)	27.6(18.9-40.1)	136.6(73.9-252.6)	16.8(14.7-19.1)
Delta						
Seroconversion	0	12(70.6,46.6-94.7)	1(8.3, -10-26.7)	5(27.8,4.9-50.7)	0	0
GMT	15.0(15.0-15.0)	32.63(24.1-44.17)	16.11(13.96-18.19)	20.3(15.6-26.4)	15.0(15.0-15.0)	15.0(15.0-15.0)
Omicron						
Seroconversion	0	0	1(7.7, -9.1-24.5)	0	0	0
GMT	15.0(15.0-15.0)	15.0(15.0-15.0)	15.94(13.96-18.19)	15.0(15.0-15.0)	15.0(15.0-15.0)	15.0(15.0-15.0)

Data are n (95% CI) for GMT, number of participants (%, 95% CI [%]) for seroconversion. CI, Confidence interval; GMT, geometric mean titer; N, Number of participants included in each group for the immunogenicity analysis.

### 3.5 CD4 and CD8 T cell responses at the V1 post-vaccination with 5 different vaccines.

**Figure 3:**
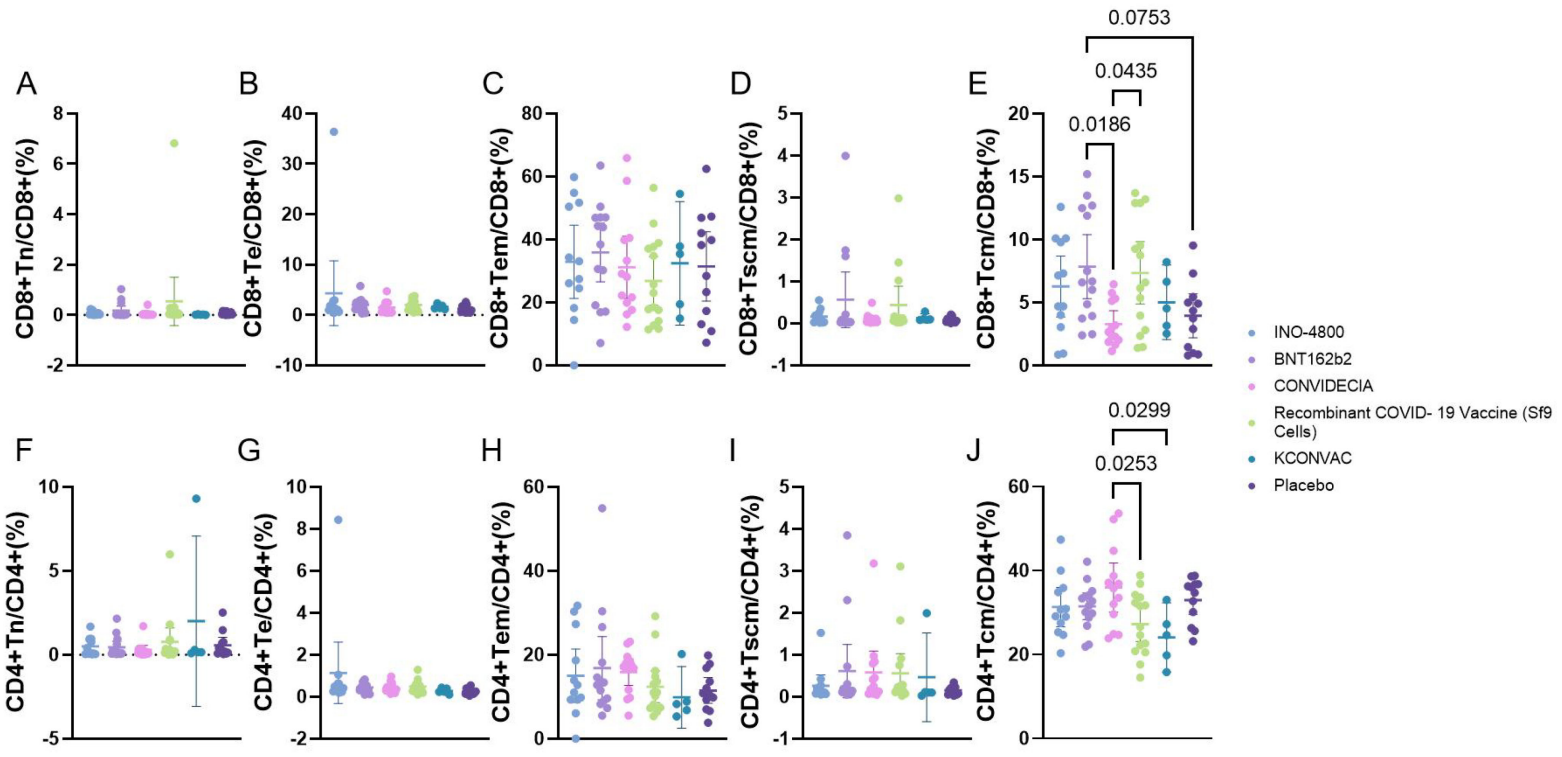
Flow cytometry analysis of the proportion of CD4/CD8 T cells at V1 with different vaccines. P<0.1 was used as the threshold for statistical significance in the analysis. **(A–E)** represent the proportions of various subgroups of cells in CD8 cells. **(F–J)** represent the proportions of various subgroups of cells in CD4 cells.

Flow cytometric analysis of the proportions of Naive T cells (Tn), Effector T cells (Te), effector memory T cells (TEM), memory stem T cells (Tscm), central memory T cells (Tcm) revealed that, except for Tcm, there were no statistically significant differences among the various vaccines. The CD8+Tcm cells were higher in the BNT162b2 group (7.862 ± 4.414) than in the CONVIDECIA vaccine group (3.298 ± 1.755), as well as higher than the Placebo group (3.953 ± 2.736) (P<0.1). Additionally, CD8+Tcm cells were higher in the Recombinant COVID-19 Vaccine (Sf9 Cells) group (7.362 ± 4.467) compared to the CONVIDECIA group (P<0.1). The CD4+Tcm cells were higher in the CONVIDECIA group (35.97 ± 9.651) than in the Recombinant COVID-19 Vaccine (Sf9 Cells) group (27.24 ± 7.461) and also higher than the KCONVAC vaccine group (24.08 ± 6.642) (P<0.1) (**Figure 3**).

### 3.6 B cell responses at the V1 post-vaccination with 5 different vaccines

**Figure 4:**
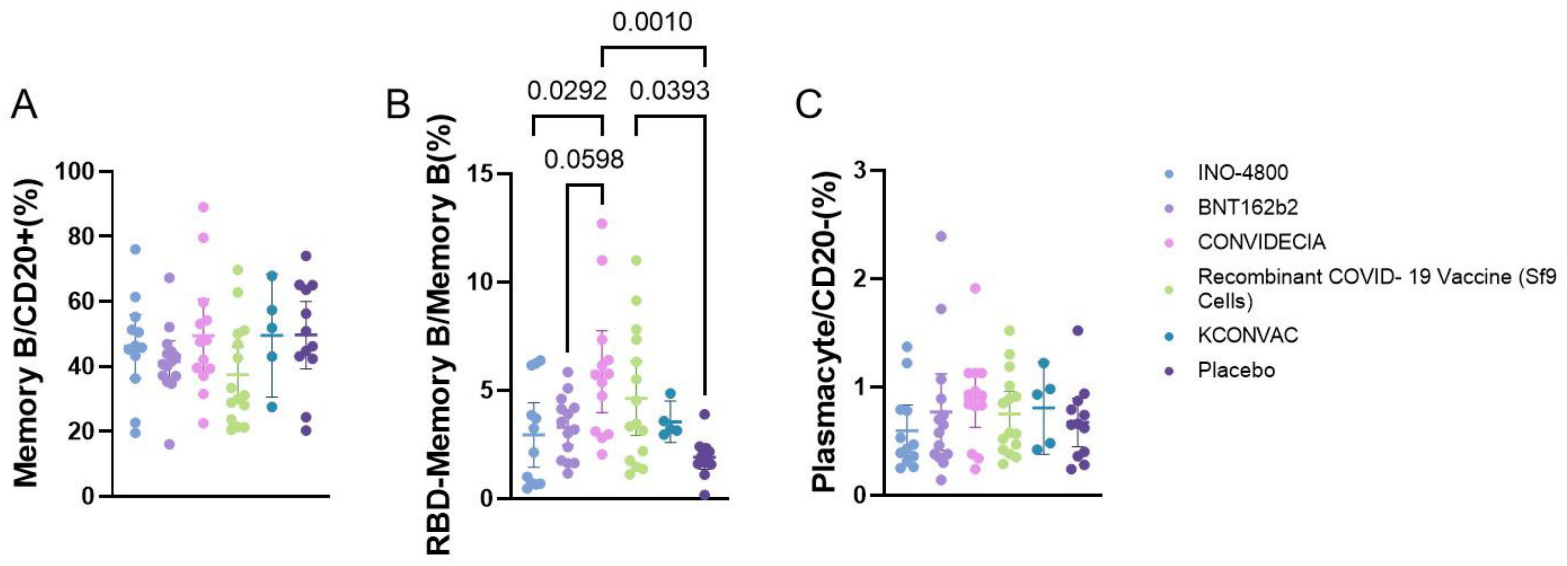
Flow cytometry analysis of the proportion of B cells at V1 with different vaccines. P<0.1 was used as the threshold for statistical significance in the analysis. **(A)** The proportion of memory B cells among CD20+ cells. **(B)** The proportion of RBD+ memory B cells among memory B cells. **(C)** The proportion of plasma cells among CD20- cells.

Flow cytometric analysis of the proportions of memory B cells, plasmacytes, and RBD-specific B cells showed that memory B cells and plasmacytes did not differ among the vaccine groups. The RBD-specific memory B cells were higher in the CONVIDECIA vaccine group (5.854 ± 3.124) compared to the INO-4800 group (2.938 ± 2.342), and higher than the BNT162b2 group (3.294 ± 1.435) and the control group (1.911 ± 0.8782). The Recombinant COVID-19 Vaccine (Sf9 Cells) group (4.632 ± 3.101) had higher levels of RBD-specific memory B cells compared to the control group (1.911 ± 0.8782) (P<0.1) (**Figure 4**).

## 4 Discussion

The COVID-19 vaccines have achieved extraordinary success in preventing infection and disease, but there are some limitations in the research on differences in immune responses between different COVID-19 vaccines, including variances in immune cells and differences in resistance to specific antigens over a certain period of time (6). This study conducted RBD-IgG binding antibody, pseudovirus neutralizing antibody, and flow cytometry experiments on INO-4800, BNT162b2, CONVIDECIA, recombinant COVID-19 vaccine (Sf9 cells), and KCONVAC to investigate the protective effects of each vaccine against human COVID-19 infection. One advantage of this study is that samples from different vaccine cohorts were subjected to the same blood processing procedures and analyzed simultaneously using the same experimental platform.

In this study, only the BNT162b2, Recombinant COVID-19 Vaccine (Sf9 Cells), and KCONVAC vaccines induced 100% individual binding antibody responses and neutralizing antibody responses at V1. The RBD-IgG GMC and neutralizing antibody GMT levels detected in the mRNA vaccine (BNT162b2) at three post-vaccination visits (V1, V2, V3) were mostly at the highest levels among the five vaccines. This is consistent with other published data indicating high to moderate antibody responses after receiving either BNT162b2 or mRNA-1273 vaccines (7). At V1, the sequence of neutralizing antibody titers (WT) for the five vaccines was BNT162b2 (743.2 (531.7-1038.7) > KCONVAC (136.6 (73.9-252.6) ~ CONVIDECIA (104.5 (57.8-189.1) > Recombinant COVID-19 Vaccine (Sf9 cells) (27.6 (18.9-40.1) ~ INO-4800 (39.7(22.8-69.1). We noted that, except for the BNT162b2 vaccine, the neutralizing antibody levels induced by the widely used KCONVAC and CONVIDECIA vaccines in China were also quite high. The differences in neutralizing antibody GMT between the two vaccines were not statistically significant at V1 and V2, but at V3, KCONVAC was lower than CONVIDECIA (P=0.026).

Additionally, we found that the neutralizing antibody titers of different vaccines are not necessarily correlated with RBD-IgG concentrations (**Supplementary Figures S1A-C**), but the trend of change is generally consistent (**Supplementary Figures S1D-F**). ELISA is one of the most commonly used detection methods in the field of immunology, known for its sensitivity, simplicity, safety, and low cost. In this study, an indirect ELISA method was used to detect RBD-specific neutralizing antibody levels targeting the RBD region of the S protein. Pseudovirus neutralization assays were carried out by expressing the S protein on the surface of pseudoviruses. Furthermore, the production principles of different vaccines also vary. For instance, BNT162b2 expresses RBD, the CONVIDECIA vaccine expresses the S protein, inactivated vaccines use whole virus inactivation, the INO-4800 vaccine expresses the S protein, and the Recombinant COVID-19 Vaccine (Sf9 cells) contains S-RBD complexes, thus the immunological effects are also influenced by the different vaccine expression and delivery systems. Moreover, we observed that once the RBD-IgG GMC decreases to a certain level, the decline in pseudovirus neutralizing effect is faster compared to the decrease in RBD-IgG GMT (**Supplementary Figure S1**). In conclusion, RBD-specific antibodies and neutralizing antibodies are both important indicators reflecting protective effects. In the absence of laboratory conditions for conducting authentic virus neutralization assays, these two test results can provide researchers with valuable references.

Due to many mutations in the S protein affecting the binding of neutralizing antibodies, SARS-CoV-2 variants pose a potential threat to the effectiveness of vaccines. In this study, we demonstrated that sera from vaccinated individuals exhibited varying degrees of reduced or completely lost neutralizing abilities against five different SARS-CoV-2 variants. The Beta strain S protein carries 9 mutations (L18F, D80A, D215G, R246I, K417N, E484K, N501Y, D614G, A701V), with K417N, E484K, and N501Y located in the RBD region. E484 is an immunodominant spike residue that can evade monoclonal antibodies and convalescent plasma antibodies, and the E484K mutation has been found multiple times in SARS-CoV-2 infected individuals worldwide (8). Additionally, studies have shown that N501Y is one of the mutations in the RBD region that leads to the highest affinity with ACE2 (9, 10). Research by Wang et al. indicates that mutations in the RBD region such as K417N/T, E484K, and N501Y significantly reduce the serum neutralizing abilities against SARS-CoV-2 pseudoviruses (11). mRNA vaccines exhibit some neutralizing activity against the Beta strain (12, 13), similar to our experimental results. In this study, only mRNA vaccines showed some neutralizing activity against the Beta strain among the five vaccines tested, but the GMT of neutralizing antibodies decreased by 5.85-19.03 times compared to the wild-type strain. The P.1 strain contains mutations E484K, N501Y, and K417T, which enhance the virus’s affinity with ACE2 and enable evasion of neutralizing antibodies in convalescent plasma from early COVID-19 patients (14). In this study, at V1 and V2, INO-4800, BNT162b2, and CONVIDECIA showed no significant increase or decrease in neutralizing ability against the P.1 pseudovirus compared to the WT. Recombinant COVID-19 vaccines (Sf9 cells) V1 and KCONVAC V2 also exhibited no significant increase or decrease in immune response against the P.1 pseudovirus compared to the WT. Previous studies have shown that the Beta variant is more difficult to neutralize than the Gamma variant (12, 15), and our results also demonstrate a similar phenomenon. A possible explanation is that substitutions in the N-terminal domain (NTD) in the Beta variant contribute to neutralization escape (16), whereas mutations in other regions of the Gamma variant enhance neutralization activity, potentially compensating for the effects of the E484K/N501Y mutations (8). In general, in the V1, BNT162b2 exhibited higher neutralizing antibody GMT against the WT, Beta, Gamma, and Epsilon variants compared to the other four vaccines, and the differences were statistically significant.

The characteristics of the Delta variant include mutations in the spike protein at positions T19R, Δ157-158, L452R, T478K, D614G, P681R, and D950N. Omicron has several deletions and over 30 mutations, some of which (69-70del, T95I, G142D/143-145del, K417N, T478K, N501Y, N655Y, N679K, and P681H) overlap with mutations found in the alpha, beta, gamma, or delta variants. The Omicron variant evades neutralization by antibodies from convalescent individuals or those vaccinated with BNT162b2, with an evasion efficiency 12-44 times higher than the Delta variant (17). A study published online in Science on February 15th regarding COVID-19 vaccine protection suggests that over time, vaccine effectiveness diminishes, and six months after two doses, the vaccines’ protection against Delta and Omicron strains is essentially lost (18). These findings are similar to ours. Results from pseudovirus neutralization assays show that BNT162b2, CONVIDECIA, and the recombinant COVID-19 vaccine (Sf9 cells) exhibit positive reactions against the Delta strain with GMTs of 32.63, 16.11, and 20.3, respectively. Compared to the WT strain, the reduction is 22.78-fold, 6.49-fold for BNT162b2 and CONVIDECIA, respectively, with no statistically significant differences. Only CONVIDECIA shows a serum conversion rate of 7.7% against the Omicron variant, while the neutralizing titers of other vaccines against Omicron pseudovirus are below the detection limit.

Some studies have shown that at the same time point after vaccination, RBD-specific MBCs in mRNA-1273~BNT162B2 > Ad26.COV2.S~NVX-CoV2373 (6). Our research results indicate that CONVIDECIA and Recombinant COVID-19 vaccines (Sf9 cells) induce higher levels of RBD-MBCs, and the expression level of RBD-MBCs in CONVIDECIA is superior to INO-4800 and BNT162b2 vaccines. There are fewer studies comparing non-specific T cell responses among different vaccines. Some studies suggest that the more severe the symptoms during hospitalization, the fewer CD4+Tcm cells in the peripheral blood during the recovery period (19). Our research shows that different vaccines injections can lead to differences in the proportion of CD4 or CD8 TCM cells, with CD4+TCM cells being highest in BNT162B2 and CD8+TCM cells being highest in CONVIDECIA.

Currently, the main types of vaccines being developed globally include nucleic acid vaccines, inactivated vaccines, non-replicating/replicating viral vector vaccines, and others. The initial COVID-19 vaccines were designed against the S protein of the SARS-CoV-2 virus. Due to factors such as the transmissibility of variant strains, population immunity levels, and public health measures, it is challenging to assess the effectiveness of SARS-CoV-2 vaccines in the real world. Although vaccine efficacy can be evaluated in clinical trials, it is difficult to directly compare different vaccines due to variations in clinical trial locations, study populations, circulating viral strains during the trials, and differences in clinical study endpoints. Among the five vaccines we studied, the mRNA vaccine (BNT162b2) demonstrated relatively strong neutralizing ability, but its neutralizing activity against the beta variant and Omicron variant also significantly decreased. The development of SARS-CoV-2 vaccines has brought hope to combating the pandemic, but the continuous evolution of this virus makes this hope uncertain. Given the immune evasion capability of the Omicron variant, it is necessary to further monitor the viral mutation patterns. More research is needed to evaluate the causes of breakthrough infections and analyze the relationship between site mutations and vaccine efficacy decline (20). There are many methods to enhance vaccine effectiveness, such as using heterologous sequence vaccine administration schedules, nasal spray immunization, or developing bivalent COVID-19 vaccines. In summary, our data indicate that there are major differences in the magnitude of functional antibody responses stimulated by the five vaccines studied and suggest that additional public health interventions such as booster vaccine doses, potentially with the more potent vaccine types, may be needed to further control the COVID-19 pandemic in worldwide. However, faced with the public health crisis of increasing SARS-CoV-2 infections and limited supply or distribution of the most effective vaccines, widespread vaccination with a lower-efficacy vaccine may still represent a route to decreasing infections, hospitalizations, and mortality.

This study has several limitations. Firstly, the study primarily used SARS-CoV-2 variant pseudoviruses. Although research has shown good consistency between pseudovirus neutralization assays and live SARS-CoV-2 virus neutralization assays, there may be differences in consistency between different strains. Additionally, the analysis of cellular immunity levels in this study was conducted at 1-2 months post-vaccination, which is a relatively short timeframe, and some differences in cellular immunity may not have been detected. Third, of course, the vaccines used in this study are all designed for the original strain of the COVID-19 virus, which prevents us from comparing the neutralization differences against different variants for vaccines designed for those variants.

## Data Availability

The raw data supporting the conclusions of this article will be made available by the authors, without undue reservation.
