# Supplementary material for: A head-to-head comparison of humoral and cellular immune responses of five COVID-19 vaccines in adults in China
Source: Front Immunol. 2024 Aug 21;15:1455730. doi: 10.3389/fimmu.2024.1455730 (PMC11371563; doi:10.3389/fimmu.2024.1455730)
Supplement: Supplementary file 1 [file DataSheet1.docx]

Supplementary Material

# Supplementary Data

Appendix 1. Inclusion and exclusion criteria.

Inclusion criteria

1．Aged 6-59 years.

2．Able to understand the content of informed consent and willing to sign the informed consent

3．Able and willing to complete all the scheduled study process during the whole study follow-up period (about 12 months).

4．Axillary temperature ≤37.0°C

5．Negative serum IgM and IgG to the SARS-CoV-2

6．Not vaccinated any type of SARS-CoV-2

7．No history of epidemiological exposure to COVID-19; No travel history to medium or high risk areas in the past 21 days;

8．General good health as established by medical history and physical examination

Exclusion Criteria

1.Family history of seizure, epilepsy, brain or mental disease

2.Participant that has an allergic history to any ingredient of vaccines

3.Woman who is pregnant, breast-feeding or positive in pregnancy test on day of enrollment, or is planning to be pregnant during the next 12 months

4.Any acute fever disease or infections or have a medical history of SARS

5.Have serious cardiovascular diseases, such as arrhythmia, conduction block, myocardial infarction, severe hypertension and not well-controlled

6.Major chronic illness, such as asthma, diabetes, or thyroid disease, and not well-controlled

7.Hereditary angioneurotic edema or acquired angioneurotic edema

8.Urticaria in last one year - Asplenia or functional asplenia

9.Asplenia or functional asplenia

10.Platelet disorder or other bleeding disorder may cause injection contraindication

11.Faint at the sight of blood or needles.

12.Prior administration of immunodepressant or corticosteroids, antianaphylaxis treatment, cytotoxic treatment in last 6 months

13.Prior administration of blood products in last 4 months

14.Prior administration of other research medicines in last 1 month

15.Prior administration of attenuated vaccine in last 1 month

16.Prior administration of subunit vaccine or inactivated vaccine in last 14 days

17.Being treated for tuberculosis

18.A history of COVID-19 infection/illness

19.Any condition that in the opinion of the investigators may interfere with the evaluation of study objectives

# Supplementary Figures and Tables

## Supplementary Figures


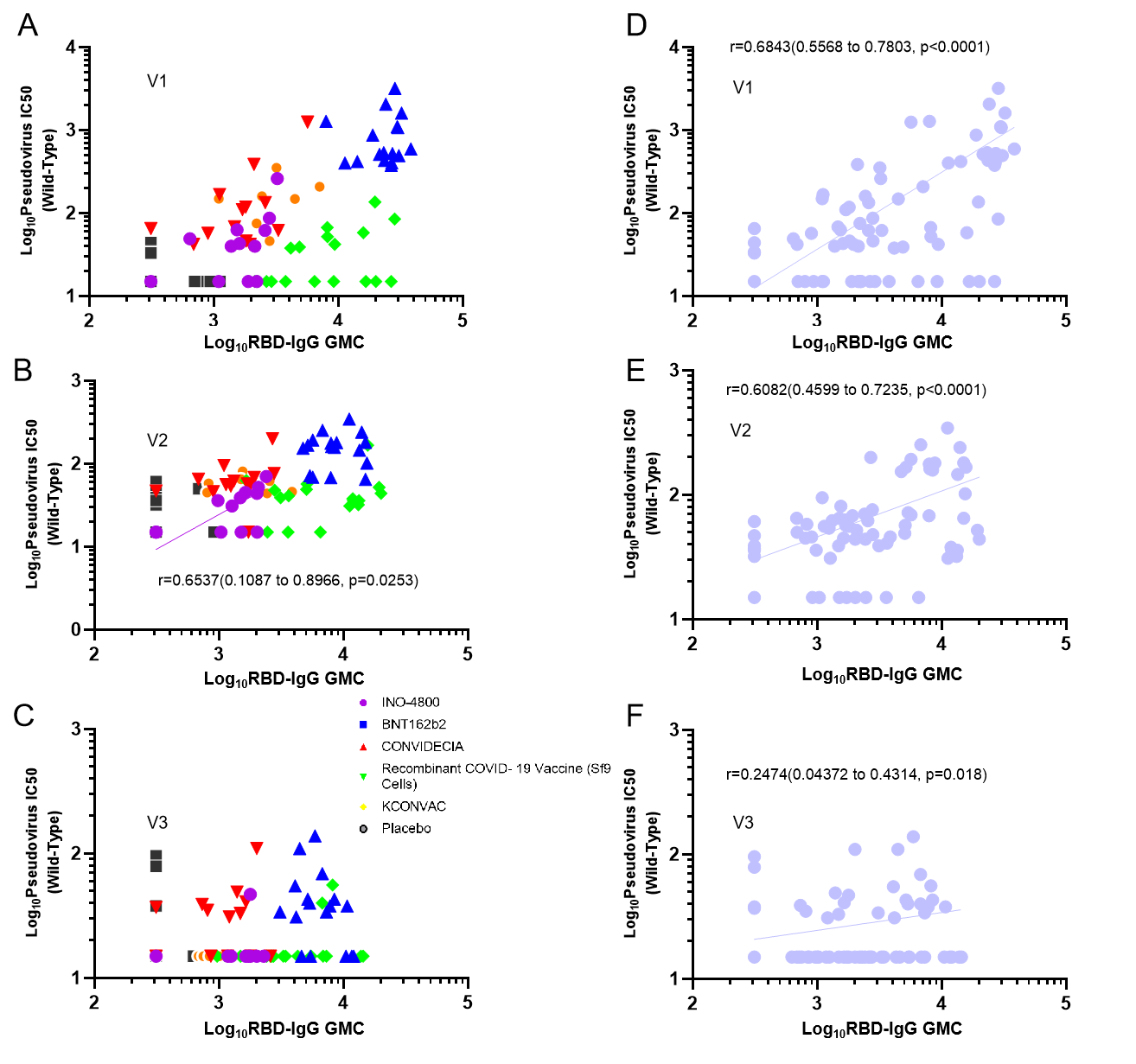


**Figure S1** Vaccine-specific immunological correlations analyses

(A-C) The association of indicated parameters shown by scatterplot. INO-4800 (purple), BNT162b2 (blue), CONVIDECIA (red), Recombinant COVID-19 Vaccine (Sf9 Cells) (green), KCONVAC (yellow) and Placebo (black). Pearson correlation coefficients (95% CIs) and p values were shown. (A)-V1, (B)-V2, (C)-V3.

(D-F) Pearson linear correlation between Log10 (Pseudovirus neutralizing antibodies titer IC50) (Wild-Type) and Log10 (RBD IgG GMC) of all vaccine at V1, V2 and V3. Pearson correlation coefficients (95% CIs) and p values were shown.


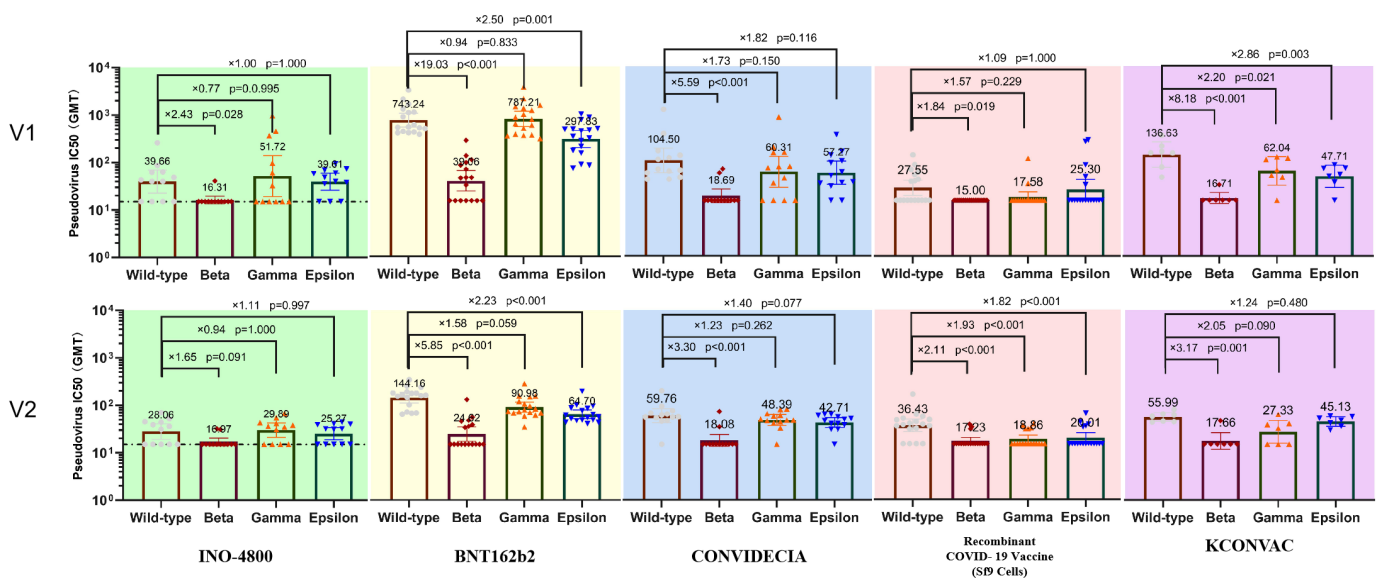


**Figure S2** Pseudovirus neutralization results of different vaccines (VOC or VOI compared with wild strain (WT)), The first row is the result at time V1, and the second row is the result at time V2. p values show differences between the different variant. GMT, geometric mean titers. Top bars indicate fold changes between two time points.


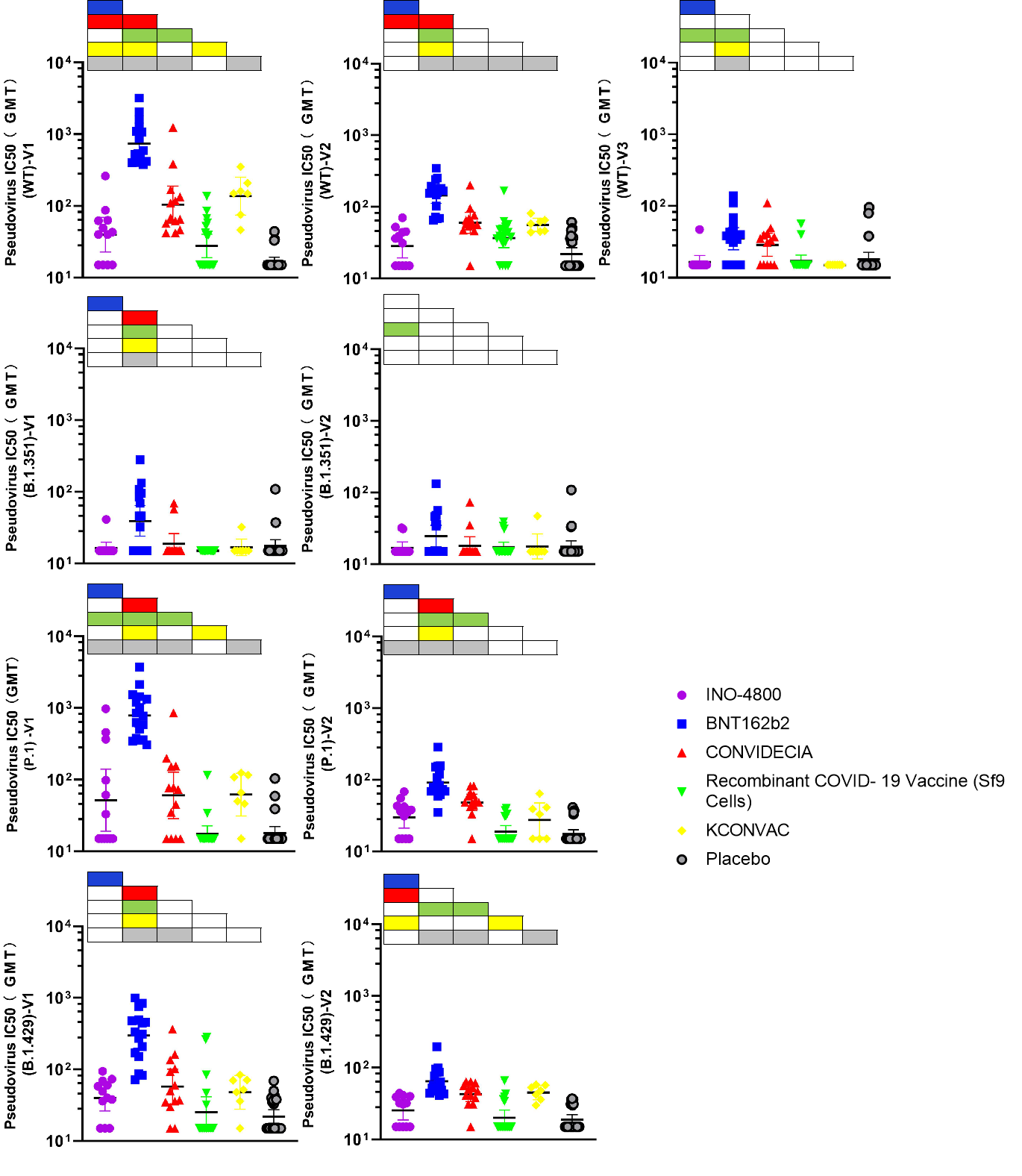


**Figure S3** Pseudovirus neutralization results of different variant (among different vaccines), The first row is the result of WT, the second row is the result of Beta variant (B.1.351), the third row is the result of Gamma variant (P.1) and the fourth row is the result of Epsilon variant (B.1.429). Inter-group comparisons with statistical significance were indicated by the colors in the boxes, with different colors representing comparisons with corresponding vaccines. Blank spaces indicate no statistical significance among groups.


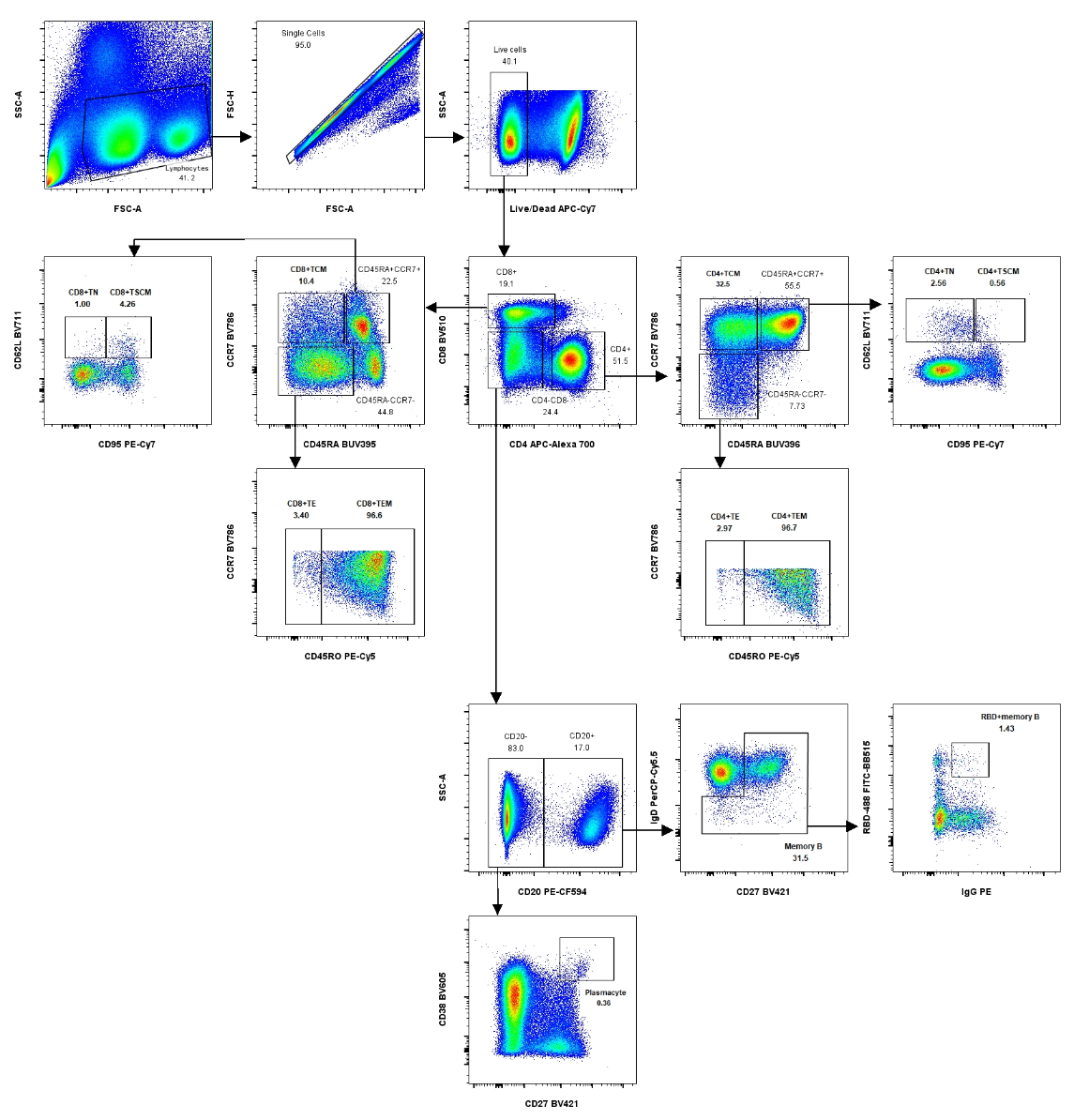


**Figure S4** Flow cytometry gating strategy for identification of T and B cells.

The flow antibody was labeled as follows: Naive T cell (Tn, CD45RA^+^CCR7^+^CD62L^+^CD95^-^), T memory stem cell (Tsc, CD45RA^+^CCR7^+^CD62L^+^CD95^+^), Central memory T cell (Tcm, CD45RA^-^CCR7^+^), Effector T cell (Te, CD45RA^-^CCR7^-^CD45RO^-^), Effector memory T cell (Tem, CD45RA^-^CCR7^-^CD45RO^+^), Memory B (MBC, CD4^-^CD8^-^CD20^+^IgD^+^CD27^+^/^-^), RBD-Memory B (RBD-MBC, CD4^-^CD8^-^CD20^+^IgD^+^CD27^+^/^-^IgG^+^RBD^-^488^+^), Plasmacyte (CD4^-^CD8^-^CD20^-^CD38^+^CD27^+^).

## Supplementary Table

Table S1 Proportion of each cell category in each vaccine

| Items | INO-4800 | BNT162b2 | CONVIDECIA | Recombinant COVID- 19 Vaccine (Sf9 Cells) | KCONVAC | Placebo |
| --- | --- | --- | --- | --- | --- | --- |
|  | (n=12) | (n=14) | (n=13) | (n=15) | (n=14) | (n=12) |
| Memory B/CD20+ | 46.15±15.43 | 41.45±11.1 | 49.48±18.42 | 37.43±15.61 | 49.54±15.27 | 49.68±16.34 |
| RBD memory B/memory B | 2.938±2.342 | 3.294±1.435 | 5.854±3.124 | 4.632±3.101 | 3.55±0.7671 | 1.911±0.8782 |
| Plasmacyte/CD20- | 0.5967±0.3718 | 0.7679±0.6128 | 0.8892±0.4298 | 0.75±0.38 | 0.806±0.3436 | 0.6725±0.3521 |
| CD8+TN | 0.07799±0.07861 | 0.1689±0.3156 | 0.05394±0.1081 | 0.5432±1.739 | 0.01721±0.01262 | 0.07145±0.06377 |
| CD8+TSCM | 0.163±0.1606 | 0.5661±1.146 | 0.1108±0.1239 | 0.4363±0.8149 | 0.1292±0.08457 | 0.08552±0.06251 |
| CD8+TCM | 6.283±3.828 | 7.862±4.414 | 3.298±1.755 | 7.362±4.467 | 5.028±2.382 | 3.953±2.736 |
| CD8+TE | 4.328±10.13 | 2.001±1.351 | 1.498±1.25 | 1.966±1.179 | 1.61±0.4901 | 1.222±0.6804 |
| CD8+TEM | 32.89±18.22 | 35.89±16.09 | 31.15±16.34 | 26.83±13.95 | 32.4±15.83 | 31.4±17.31 |
| CD4+TN | 0.5028±0.5415 | 0.4397±0.6287 | 0.2935±0.4472 | 0.7731±1.514 | 2±4.081 | 0.5668±0.7418 |
| CD4+TSCM | 0.2631±0.4095 | 0.6101±1.1 | 0.5807±0.8425 | 0.5569±0.8482 | 0.4658±0.8527 | 0.1502±0.09258 |
| CD4+TE | 1.138±2.307 | 0.4429±0.218 | 0.4269±0.2377 | 0.486±0.2877 | 0.28±0.13 | 0.2411±0.1348 |
| CD4+TEM | 15.02±9.981 | 16.82±13.03 | 15.88±5.261 | 12.39±6.812 | 9.898±5.92 | 11.5±4.828 |
| CD4+TCM | 31.31±7.353 | 31.49±5.501 | 35.97±9.651 | 27.24±7.461 | 24.08±6.642 | 32.98±5.394 |
